# A scoping review on disparities in exposure to advertising for e-cigarettes and heated tobacco products and implications for advancing a health equity research agenda

**DOI:** 10.1186/s12939-021-01576-2

**Published:** 2021-10-30

**Authors:** Graziele Grilo, Elizabeth Crespi, Joanna E. Cohen

**Affiliations:** Institute for Global Tobacco Control, Department of Health, Behavior and Society, Johns Hopkins Bloomberg School of Public Health, 2213 McElderry Street, Fourth Floor, Baltimore, MD 21205 USA

**Keywords:** E-cigarette, Heated tobacco products, Tobacco advertising and promotion, Health equity, Tobacco-related disparities

## Abstract

**Background:**

Disparities in exposure to and density of tobacco advertising are well established; however, it is still unclear how e-cigarette and heated tobacco product (HTP) advertising vary by age, education, sex, gender identity, race/ethnicity, sexual orientation, socioeconomic status (SES), and/or urban/rural area. Through a scoping review, we sought to identify potential disparities in exposure to e-cigarette and HTP advertising and promotion across populations.

**Methods:**

In January 2020, a systematic literature search was conducted in five databases: PubMed, Scopus, Embase, Web of Science, and the Cochrane Library. The search was updated in October 2020. Articles reporting on exposure to e-cigarette and/or HTP advertising and promotion across age, education, sex, gender identity, race/ethnicity, sexual orientation, SES, and/or urban/rural areas were included for full-text review (*n* = 25). Of those, 15 were deemed relevant for data extraction.

**Results:**

The majority of the studies were from the U.S. (*n* = 12) and cross-sectional (*n* = 14). Studies were published between 2014 and 2020 and focused on determining causal relationships that underlie disparities; only one study assessed HTP advertising and promotion. Exposure to e-cigarette and HTP advertising was assessed at the individual-level (e.g., recall seeing ads on television) and at the neighborhood-level (e.g., ad density at the point-of-sale). Studies addressed differences across age (*n* = 6), education (*n* = 2), sex (n = 6), gender identity and sexual orientation (*n* = 3), race/ethnicity (*n* = 11), SES (*n* = 5), and urban/rural (n = 2). The following populations were more likely to be exposed to e-cigarette advertising: youth, those with more than a high school diploma, males, sexual and gender minorities, Whites, and urban residents. At the neighborhood-level, e-cigarette advertisements were more prevalent in non-White neighborhoods.

**Conclusions:**

Exposure to e-cigarette/HTP advertising varies based on sociodemographic characteristics, although the literature is limited especially regarding HTPs. Higher exposure among youth might increase tobacco-related disparities since it can lead to nicotine/tobacco use. Research should incorporate and apply a health equity lens from its inception to obtain data to inform the elimination of those disparities.

## Introduction

Factors such as housing, discrimination, employment, and education have been shown to impact individual and population-level health, resulting in systematic and avoidable health disparities among certain populations [1–3]. Tobacco use, for example, disproportionately affects those living in poverty, suffering from mental illness, and with lower educational attainment [4]. In fact, smoking contributes substantially to health disparities; low socioeconomic status (SES) populations have higher smoking rates [5, 6] and greater smoking-related mortality [7]. Differences in mortality rates between those of high- and low-SES have been linked largely to smoking [8]. Previous research has shown that the tobacco industry has targeted populations that have been excluded or marginalized; a systematic review of the literature found elevated tobacco advertising in lower income neighborhoods [9]. Additionally, menthol advertising is more prevalent in low-SES, urban, and disproportionately Black neighborhoods [9, 10]. Laws et al. found a higher proportion of businesses with storefront tobacco advertising in predominantly Black or Latino, low-SES neighborhoods [10]. Evidence suggests the tobacco industry creates product brand identities targeting certain populations [11]. This purposive targeting is concerning, as evidence has shown that tobacco advertising can reinforce beliefs that tobacco use is normal [11, 12]. Moreover, several studies have linked advertising exposure to smoking initiation, susceptibility to smoking, and smoking behaviors, leading to a 2012 declaration by the U.S. Surgeon General that exposure to tobacco advertising causes smoking initiation [13].

The elimination of these disparities by addressing the social determinants of health is necessary in achieving health equity, in which “everyone has a fair and just opportunity to be as healthy as possible” [14]. However, there is a need to better understand the pathways through which social determinants influence health and the effectiveness of interventions that address these determinants in improving health for all populations [3]. Research can support this goal by documenting, understanding, and addressing disparities. Thomas et al. provide a framework for advancing health equity through research [15]. The Health Equity Action Research Trajectory (HEART) paradigm categorizes studies into four research generations: (1) document existing disparities; (2) determine causal relationships that underlie disparities; (3) identify solutions for eliminating disparities using transdisciplinary research, community engagement, and translational research; and (4) take action to eliminate disparities by using public health critical race praxis as a conceptual framework, addressing structural determinants of health through multilevel interventions, using comprehensive evaluation, and engaging in self-reflection as researchers [15]. The framework is also useful for evaluating gaps in the current literature because it identifies necessary steps for advancing a health equity research agenda by categorizing research in each generation [15].

The recent emergence of e-cigarettes and heated tobacco products (HTPs) has raised concerns about how they might increase/decrease tobacco-related disparities. Similar concerns have also been raised regarding advertising exposure, including that these products may renormalize cigarette smoking [16, 17]. E-cigarettes are electronic devices that heat a liquid to produce an aerosol for inhalation, which often contains nicotine and flavorings, but can also contain THC, CBD, vitamins, and other additives. Though the industry has marketed e-cigarettes as smoking cessation and harm reduction tools [18], research on their effectiveness for smoking cessation is mixed [19, 20].

HTPs produce an aerosol by electronically heating tobacco [21]. HTPs have been marketed as less harmful alternatives to combustible tobacco products [22], though these devices still produce harmful emissions [23] and further research is needed to understand their health effects [24].

In 2018, the tobacco industry spent $110 million on e-cigarette advertising in the U.S. alone [25]. This same year, the U.S. Surgeon General declared that e-cigarette use among youth was an epidemic due to its rapid increase among middle and high-school students [26]. Evidence suggests that e-cigarette advertising influences perceptions of and interest in e-cigarettes among adolescents [27]. A study found that e-cigarette advertising in the U.S contained youth-appealing features [28]. Research has also shown that e-cigarette use may result in transitions to cigarette smoking [29], leading to further concerns about how these devices may affect existing disparities in health outcomes related to tobacco. Current research in this regard has not been fully explored; a systematic review found different patterns for e-cigarette awareness, ever use, and current use based on sociodemographic characteristics, highlighting the need to ensure the potential benefits and risks of e-cigarettes do not increase current health disparities [30]. Two other systematic reviews examined the potential equity impact of non-combustible nicotine products, including e-cigarettes, for cigarette smoking cessation/reduction [31] and smokers’ engagement with these products [32] among smokers from different SES. These studies found little evidence of the impact of e-cigarettes on cigarette smoking disparities [31] and a lack of studies on higher-SES groups [32].

It remains unknown how these devices are currently being advertised to various populations, if exposure level varies across populations, and how this might impact tobacco-related disparities. This paper aims to fill this gap by answering the following questions: (1) According to the current literature, how does e-cigarette/HTP advertisement exposure differ across race/ethnicity, age, education, sex, gender identity, sexual orientation, SES, and/or urban/rural areas? (2) In which generations of the HEART framework can the available literature be categorized? Understanding the disparities that exist in e-cigarette/HTP advertising and gaps in the literature may guide future research and allow for more effective action on eliminating tobacco-related disparities and advancing health equity.

## Methods

In order to map the existing literature focusing on advertisement exposure across different sociodemographic characteristics, we conducted a scoping review using the steps proposed by the Arksey and O’Malley framework [33]. After 1) identifying our research questions, we 2) identified the relevant studies; 3) applied inclusion/exclusion criteria; 4) extracted the data; and, 5) collated, summarized, and reported the results. This analysis is part of a larger study in which the final step, 6) a consultation with experts, was also conducted; however, we do not report the results here because the consultation focused on research priorities related to e-cigarettes, HTPs, and tobacco-related disparities in relation to prevalence, susceptibility, cessation, access, and, advertising [34]. For this study, we focus on the results related to advertising.

### Identifying relevant studies

The initial search was conducted on January 14, 2020 in five electronic databases (PubMed, Scopus, Embase, Web of Science, and the Cochrane Library), which were determined in consultation with a university informationist who also supported the construction of the search strategy (Table 1). We updated our search on October 19, 2020 using the same strategy and databases. Results were uploaded to Covidence, which removed duplicates automatically. In addition, one study was recommended by a convening participant.

**Table 1:** Search terms

#1	Electronic Nicotine Delivery Systems[mesh] OR Vaping[mesh] OR Electronic Cigarette*[tw] OR E-Cigarette*[tw] OR E-Cig*[tw] OR Vape[tw] OR vaping[tw] OR vaper*[tw] OR JUUL[tw] OR IQOS[tw] OR heat-not-burn[tw] OR heated tobacco products[tw] OR JUULing[tw]
#2	ethnic groups[mesh] OR Socioeconomic Factors[mesh] OR Social Class[mesh] OR Age Factors[mesh] OR Sex Factors[mesh] OR Race Factors[mesh] OR Educational Status[mesh] OR health equity[mesh] OR health status disparities[mesh] OR Socioeconomic Factor*[tw] OR Inequalit*[tw] OR Sex Factor*[tw] OR Sexualit*[tw] OR Race Factor*[tw] OR Age Factor*[tw] OR sociodemographic*[tw] OR Socioeconomic Status[tw] OR Social Classes[tw] OR health equities[tw] OR Race[tw] OR Adolescen*[tw] OR Youth*[tw] OR Teen*[tw]
#3	#1 AND #2

### Applying inclusion/exclusion criteria

To meet our inclusion criteria, studies had to be 1) related to e-cigarettes and/or HTPs; and, 2) related to health equity and/or disparities. For the purposes of this paper, we also excluded studies that did not report on e-cigarette/HTP advertising. During this step, studies were also classified based on three priorities: high – clear discussion of the implications of e-cigarettes/HTPs in relation to health equity/disparities; mid – no discussion of implications, but more than just presenting descriptive information about advertising exposure by different populations OR studies that present unique data or perspective on the issue; and, low – only descriptive information on e-cigarette/HTP advertising exposure by different sociodemographic characteristics. Titles and abstracts were double-coded by independent researchers (GG, EC, AA). Disagreements related to inclusion/exclusion and/or priority setting were resolved by consensus; a fourth researcher (JEC) made the final decision when consensus was not reached.

### Extracting data

Only high and mid priority studies were eligible for full text review due to time constraints. Two researchers (GG and EC) reviewed an initial set of studies to refine data extraction procedures. Remaining studies were individually reviewed; however, excluded studies were reviewed for agreement. The following information was extracted using Microsoft Excel: a) citation; b) objective; c) study design; d) study population; e) key results and conclusion; f) policy/research implications; g) main theme; h) product type (e-cigarettes and/or HTPs); i) funding; j) research generation; k) notes.

### Collating, summarizing, and reporting the results

Both researchers (GG and EC) reviewed the extracted information and categorized the studies based on the HEART paradigm. Since we were interested in assessing differences by sociodemographic characteristics, key results and conclusions were then summarized by age, education, sex, gender identity, sexual orientation, race/ethnicity, SES, and urban/rural. Results are presented by subpopulation.

## Results

### Results of the search

**Fig. 1:**
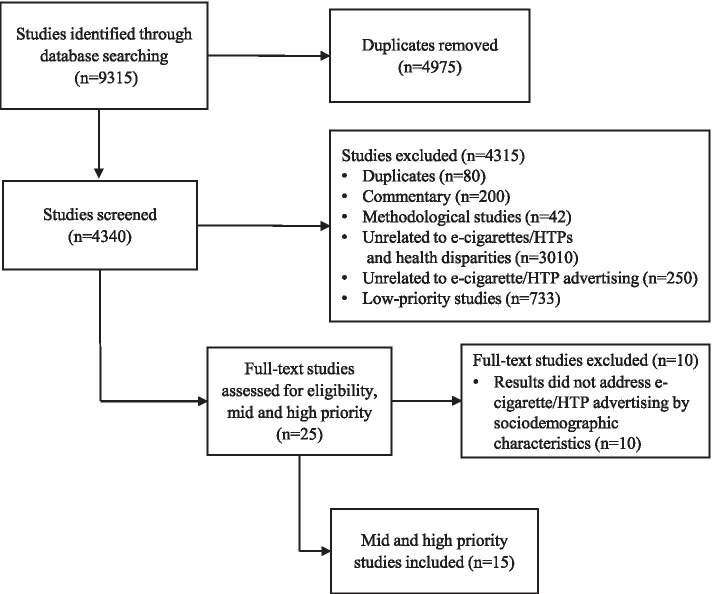
Flowchart of included studies

A total of 15 studies were identified. Figure 1 illustrates the study selection process.

### Characteristics of included studies

Of the 15 included studies, 12 were from the U.S. (six were nationally representative, but one only in the contiguous U.S.); one was from the U.S. and Canada; one from Guatemala; and, one did not have a specific location (i.e., the Internet). Fourteen studies were cross-sectional, and one was longitudinal. All studies were categorized as in the second generation of the HEART paradigm (determining causal relationships that underlie disparities) [15].

Exposure to e-cigarette advertising and promotion by sociodemographic characteristic was measured at the individual level, with some studies also assessing different channels of exposure, and at the neighborhood level, focusing on different points-of-sale (POS). One study focused on the content of e-cigarette advertising in magazines and another one on Twitter. Only one study assessed HTP marketing; this study observed product placement and advertising at POS in Guatemala [35]. Table 2 provides more information about the studies in the sample.

**Table 2:** Summary of included studies

Lead author (Country, Year)	Focus	Study design	Study population	Disparities assessed by	Major Findings
Barnoya J et al. (Guatemala, 2020) [35]	E-cig/HTP POS advertising	Cross-sectional	Convenience stores in gas stations	Age, SES	(1) E-cigs and HTPs found near candy, especially in high-SES neighborhoods. (2) More e-cigs and HTPs next to the cash register in high-SES than mid-SES neighborhoods. (3) More e-cig interior ads in mid-SES than high-SES neighborhoods.
D’Angelo et al. (US, 2020) [36]	E-cig POS availability/promotions/marketing	Longitudinal	Tobacco retailers that sell cigarettes (nationally representative in the contiguous US)	Age, race/ethnicity, SES	(1) About one quarter of retailers displayed e-cigs near candy and/or sweet beverage and had interior e-cig signs/ads located at child eye level. (2) More price promotions in disproportionately Hispanic, but not disproportionately Black, neighborhoods. (3) More exterior e-cig advertising at retailers in neighborhoods with the most, compared to the least, Black residents. (4) Neither e-cig promotion nor exterior e-cig advertising prevalence associated with neighborhood median household income.
Escobedo et al. (US, 2019) [37]	E-cig availability/marketing in low-income communities	Cross-sectional	Tobacco retailers	Race/ethnicity	(1) Retailers in African American, Korean American and Hispanic/Latino communities less likely to have e-cigs placed near youth-friendly items. (2) Retailers in Korean American and Hispanic/Latino communities less likely to have exterior e-cig advertising.
Emory et al. (US, 2019) [38]	Tobacco-related content exposure/channels	Cross-sectional	Adults (nationally representative)	Gender identity and sexual orientation	(1) LGBT more likely than non-LGBT to search for, share, be exposed to e-cig content in past 30 days. (2) LGBT not more likely to be exposed to anti-tobacco content in past 30 days. (3) Past 30-day e-cig ad exposure higher in LGBT smokers. (4) LGBT less likely than non-LGBT to be exposed to e-cigs on TV, more likely to be exposed on internet and social media.
Wagoner et al. (US, 2019) [39]	E-cig advertising exposure/channels	Cross-sectional	Individuals ages 13+ (nationally representative)	Age, education, sex, race/ethnicity	(1) Compared to older adults, adolescents more often exposed to e-cig ads on TV and digitally, and less often exposed in retail, radio, and print media. (2) E-cig ad exposure highest for those with some college, then those with a 4-yr degree or more, and then those with a high school diploma or less; higher among males than females; and highest for Whites, then Blacks, and then other races.
Pike et al. (US, 2019) [40]	E-cig ads and e-cig/cigarette/cigar use	Cross-sectional	High-risk youth with poor academic performance, conduct problems, and/or extenuating life circumstances	Sex	(1) Relation between ad exposure and e-cig use stronger among females than males.
Tan et al. (US, 2019) [41]	E-cig/cigarette/cigar/smokeless ad exposure	Cross-sectional	Young adults (nationally representative)	Gender identity and sexual orientation, race/ethnicity	(1) E-cig ad exposure higher for bisexual than heterosexual and lesbian women and gay and heterosexual men than men who identified as ‘something else’. (2) Controlling for covariates, no differences found between gay or bisexual men and heterosexual men. (3) E-cig ad exposure lower for heterosexual ‘other race’ than heterosexual white women and higher for bisexual Hispanic than heterosexual non-Hispanic women.
Simon et al. (US, 2018) [42]	E-cig ad exposure and use	Cross-sectional	High school students	SES	(1) Higher ad exposure for higher SES than lower SES students.
Wan et al. (US, 2018) [43]	E-cig POS ad density	Cross-sectional	Tobacco retailers	Age, race/ethnicity, SES, urban/rural	(1) Higher e-cig POS ad density in areas with lower proportion of adolescents, higher proportion of young adults, higher proportion of Hispanics, lower proportion of Whites, and higher proportion of Blacks, lower median family income, and in urban than rural areas.
Singh et al. (US, 2016) [44]	E-cig ad exposure	Cross-sectional	Mid/high school students (nationally representative)	Age, sex, race/ethnicity	(1) Higher e-cig ad exposure for students in higher grades across all ad sources. (2) Higher e-cig ad exposure on the internet and in newspapers/magazines for females than males. (3) Higher e-cig ad exposure in retail stores for Whites than Blacks and other non-Hispanic races. (4) Higher e-cig ad exposure from TV/movies for Blacks and Hispanics than Whites.
Sowles et al. (N/A, 2016) [45]	Characteristics of vaping-related Twitter	Cross-sectional	Vaping-related advertisements on Twitter	Sex, race/ethnicity	(1) Highly White and male audiences for vaporizers and e-liquid ads compared with the Twitter median average.
Baumann et al. (US, 2015) [46]	E-cig ad exposure	Cross-sectional	Hospitalized adult cigarette smokers	Race/ethnicity	(1) E-cig ad exposure higher for Whites than Blacks. (2) Increase in e-cig ad exposure over time greater for Whites than Blacks. (3) E-cig ever use related to ad exposure for Blacks but not Whites. (4) Different sources of ads for Blacks (radio, TV) and Whites (stores, internet).
Roberts et al. (US, 2015) [47]	External tobacco POS marketing	Cross-sectional	Tobacco retailers	Race/ethnicity, urban/rural	(1) Increased e-cig promotions in higher percentage African American communities. (2) Higher POS e-cig marketing in urban Columbus than rural Ohio.
Emery et al. (US, 2014) [48]	E-cig information exposure/channels	Cross-sectional	Adults (nationally representative)	Age, education, sex, gender identity and sexual orientation, race/ethnicity, SES	(1) E-cig info exposure associated with younger age, more education, male gender, and White race. (2) E-cig ad exposure not associated with LGB status or income.
Richardson et al. (US/Canada, 2014) [49]	Non-POS non-combustible product ads	Cross-sectional	Non-POS non-combustible product ads	Sex, race/ethnicity	(1) E-cig ads printed on magazines whose audiences are mainly White males, though Blu ads printed on some magazines targeting White women. (2) Ads tended to feature White males and females above those of other races.

### Age

Six studies reported on advertising exposure by age. Despite different study populations and locations, findings predominantly showed that adolescents and young adults are more likely to be exposed to e-cigarette and HTP advertising than adults aged 26 years and older. One U.S.-nationally representative study found higher exposure to e-cigarette advertising through TV and digital sources for adolescents between the ages of 13-17 years compared to adults over 26 years old [39]. Those over 26 years old were exposed more to e-cigarette advertising through POS, radio, and print media [39] compared to the adolescents. Another nationally representative study, of U.S.-middle and high school students, found exposure to e-cigarette advertising through all platforms (POS, Internet, TV/movies, newspapers/magazines) increased as grade level increased [44]. A third nationally representative study of U.S. adults found that the odds of searching for and sharing information about e-cigarettes fell with increasing age, with adults aged 18-24 having the highest odds, followed by adults aged 25-44, 45-64, and then 65 and older [48].

The other studies in the sample observed POS in different areas: contiguous U.S. [36], Guatemala [35], and Nebraska [43]. Two of them, one representative [36] and one non-representative [35], found e-cigarette/HTP placement near candy and/or sweet beverages, an old tactic used by the tobacco industry to promote cigarettes to children. In Nebraska, the POS with high density of e-cigarette advertising were located in neighborhoods with more young adult and fewer adolescent residents [43].

### Education

Only two studies, both U.S. nationally representative studies, examined exposure to e-cigarette advertising/information by education level; both found advertisement/information exposure to be higher for those with more than a high school diploma. Wagoner et al. found e-cigarette advertisement exposure to be highest for those with some college, followed by those with a 4-yr degree or more and then those with a high school diploma or less [39]. Similarly, Emery et al. also found exposure to e-cigarette information to be associated with more education [48].

### Sex

Only six studies evaluated e-cigarette advertising by sex. Though the exact measurement, population, and advertising source varied by study, results generally found elevated exposure among males compared to females. Three of these studies were nationally representative, two of which found elevated e-cigarette advertisement/information exposure for male than female adults [39, 48]. The third found e-cigarette advertisement exposure to be higher for female than male middle and high school students on the Internet and in newspapers/magazines [44]. An examination of magazines in the U.S. and Canada found that e-cigarette print ads occurred in magazines whose audiences consisted mainly of White males (e.g. Maxim, Rolling Stone, Men’s Journal, and Playboy), but that Blu e-cigarette ads appeared in some magazines targeting White women (e.g. Star and Us Weekly) [49]. On Twitter, the audiences for vaporizers, e-cigarette liquids, and marijuana vape pens were disproportionately male compared to the Twitter median average [45]. Additionally, a non-representative U.S. study found a stronger association between e-cigarette advertisement exposure and use among female than male Californian youth with poor academic performance, conduct problems, and extenuating life circumstances [40].

### Gender identity and sexual orientation

Three nationally representative U.S. studies examined disparities in e-cigarette advertising by LGB status, generally finding elevated e-cigarette advertising exposure among sexual and gender minorities. Of these three studies, only one considered differences between transgender and cisgender individuals as well. One found that LGB status did not predict exposure to e-cigarette information among adults [48]. On the other hand, Emory et al. found that LGBT adults, including both cigarette smokers and non-smokers, were more likely than their non-LGBT counterparts to search for, share, and be exposed to e-cigarette information. They were not, however, more likely to be exposed to anti-tobacco content. Channels of e-cigarette exposure also varied: LGBT individuals were more likely to be exposed to e-cigarettes on the internet and social media, but less likely to be exposed on TV [38]. In a study of young adults, Tan et al. found higher e-cigarette advertisement exposure for bisexual than heterosexual and lesbian women and for gay and heterosexual men than men who identified as ‘something else’ when controlling for race, ethnicity, education, and tobacco product use; no differences were found in e-cigarette ad exposure between gay or bisexual men and heterosexual men [41].

### Race/ethnicity

Eleven studies reported disparities by race/ethnicity, with four studies also reporting how race/ethnicity intersects with other sociodemographic characteristics. While some studies found that Whites were more likely to be exposed to e-cigarette advertising and information at the individual level (across different populations), other studies found the opposite at the neighborhood level, with non-White areas more likely to have increased advertisement and promotion. Four studies [39, 41, 44, 48] assessing individual exposure were nationally representative and one was not [46]; all five found that Whites were more likely to be exposed to e-cigarette advertising [39, 41, 44, 46] and information [48] than other races. When intersecting race/ethnicity with sexual orientation, Tan et al. found higher exposure to e-cig advertising among heterosexual white women than heterosexual “other races” and bisexual Hispanic than heterosexual non-Hispanic women. Two studies also reported disparities when considering advertisement source of exposure: Singh et al. found that White middle and high school students were more likely to be exposed in retailers than Blacks and other non-Hispanic races/ethnicities while Blacks and Hispanics were more likely to be exposed via TV and movies. Similarly, among hospitalized cigarette adult smokers in Alabama, Blacks had greater exposure via radio or television than Whites, whose exposure was higher via stores and the Internet [46]. A study assessing vaping related advertisements on Twitter found that the audience for vaporizers and e-cigarette liquids had a higher proportion of Whites compared to the median Twitter average [45]. Similarly, one study found that e-cigarette print advertisements were more likely to feature White males and females than other races and also to be printed in magazines whose audience was mainly White [49].

Only one study assessing tobacco retailers was nationally representative (in the contiguous U.S. only) [36]. This longitudinal study found higher prevalence of exterior e-cigarette advertising in neighborhoods with a greater proportion of Blacks, and of e-cigarette price promotions among POS located in neighborhoods with a greater proportion of Hispanics. Studies in other areas found similar results: in Nebraska, higher POS e-cigarette advertising density was significantly associated with neighborhoods with a higher proportion of Hispanics [43]; and in Ohio, e-cigarette promotion was found to be higher in neighborhoods with a higher percentage of Black residents [47]. However, a study among low-income, ethnic neighborhoods in Los Angeles found higher availability of e-cigarette advertising in non-Hispanic White neighborhoods compared to Black, Hispanic, Korean-American, and American-Indian neighborhoods [37].

### SES

Five studies examined disparities in e-cigarette advertising/information exposure by SES. The two nationally representative U.S. studies [36, 48] found similar results: one found no relationship between self-report income and e-cigarette information exposure [48] and the other found no relationship between neighborhood median household income and e-cigarette promotions or exterior advertising at tobacco retailers that sell cigarettes [36]. There were two non-representative U.S. studies in our sample [42, 43]. Simon et al. found higher advertisement exposure for high than low-SES high schoolers in Connecticut [42]. Wan et al. found higher e-cigarette POS advertising density in areas of Nebraska with lower median family income [43]. One study focused on two large cities in Guatemala. While e-cigarette interior ads were found more frequently at POS in mid-SES neighborhoods, the median numbers of interior ads (for e-cigarettes and HTPs) were higher in high-SES compared to mid-SES neighborhoods [35].

### Urban/rural

Only two studies [43, 47] in the sample reported on differences based on urban/rural location. These studies observed tobacco retailers in two different US-states: Nebraska [43] and Ohio [47]. Both found higher e-cigarette advertising or marketing in urban compared to rural areas.

## Discussion

This scoping review found that e-cigarette advertisement presence and exposure differ based on several sociodemographic factors and revealed limited information on HTP advertisement exposure. Although the literature is limited, adolescents and young adults, those with more than a high school diploma, males, sexual and gender minorities, Whites, and urban residents were more likely to be exposed to e-cigarette advertising. Studies focused on the neighborhood-level found e-cigarette advertisements to be more prevalent in non-White neighborhoods. Studies assessing advertising by SES both at the individual and neighborhood level found diverging results, with two reporting more advertising/exposure for higher-SES individuals or neighborhoods, one reporting the opposite for neighborhoods, and two finding no correlation.

It is not surprising that males and Whites were more likely to be exposed to tobacco advertising than their counterparts. Tobacco use has been historically more prevalent among men [50]; moreover, an early study found that print advertisement of e-cigarettes targeted White men [49]. Patterns of e-cigarette use in urban/rural areas have been mixed [51]. A recent review of the literature also found mixed results in urban/rural areas: while urban neighborhoods were more likely to have tobacco advertising closer to schools, playgrounds, and churches, rural youth were more exposed to tobacco advertising than their urban counterparts [52]. Urban/rural differences warrant further exploration since only two studies reported on these differences; each one of these studies focused on one U.S. state and might not be generalizable to other states.

Tobacco industry practices of targeting groups that have been excluded or marginalized are well-documented, including among LGBT populations [52–54]. However, only three studies in the sample examined advertising exposure among LGBT adults. Overall tobacco use is higher among LGBT populations than among heterosexuals [55, 56]; the lack of studies among this population still is a major gap. Future research should also consider if/how the tobacco industry’s messages and approaches in advertising vary by group and how different messages are received by populations that have been excluded or marginalized; this may improve our understanding of what appeals to different groups, illuminate how the tobacco industry is using this to target populations, and aid in developing more content-appropriate tobacco control mass media campaigns.

Contradictory findings regarding race/ethnicity at the individual versus the neighborhood level warrant further investigation. While evidence suggests that advertising exposure is linked to advertising density among youth [57], this has not been examined by race/ethnicity. In addition, studies that found higher advertisement exposure among Whites collected data between 2012 and 2015 whereas studies that found the opposite collected data between 2014 and 2015, which might indicate a shift in advertising target. A similar pattern has been observed for POS density in the U.S: earlier studies found more e-cigarette retailers in primarily White neighborhoods whereas more recent studies have found increased density in neighborhoods with more racial/ethnic minorities [34]. A more recent study in New York City, however, found a higher prevalence of e-cigarette advertising in higher-income and White neighborhoods whereas lower-income and predominantly Black and Hispanic neighborhoods had a higher prevalence of other tobacco products, such as cigars and cigarillos [58]. While these findings might not be generalizable, it might indicate that e-cigarette advertising at POS is competing with other tobacco products that have been on the market for a longer time and for which prevalence is higher among Black and Hispanics. Nevertheless, our findings are concerning given the tobacco industry’s history of targeting populations that have been excluded or marginalized [9–11].

The majority of the 15 included studies reported on e-cigarette exposure by race/ethnicity (focusing on outcomes among Black, Hispanics, and Whites), followed by age and sex. Moreover, studies in our sample did not assess how sociodemographic characteristics intersect among each other, which might have resulted in different levels of exposure. An intersectional approach might elucidate complex interactions among advertisement exposure across different groups, exposing how different socioeconomic, historical and cultural contexts might be mediating those interactions. In our sample, for example, Tan et al. assessed advertising exposure of different tobacco products considering sexual orientation, sex, and race/ethnicity and found very different patterns of exposure based on intersecting identities and certain tobacco products, essential information when designing interventions.

### Strengths and limitations

Strengths of this study include our search strategy, which was not restricted to a certain timeframe, country, language, population, or study design. However, our selection criteria excluded abstracts that did not specify results on e-cigarette and/or HTPs, which might have resulted in missing relevant studies. In addition, our priority classification of the studies was solely based on our thematic interest and we did not conduct any quality appraisal - a recognized limitation of scoping reviews [33]. Given the significant and rapid changes to the e-cigarette/HTP market, our findings might not be generalizable to today (e.g., many studies in the sample collected data before JUUL’s market explosion) [59]. Though we did not limit our search strategy to a specific country, the majority of articles in our sample were U.S.-based; therefore, findings might not be generalizable to other countries. Nevertheless, as e-cigarettes and heated tobacco products become available in other markets, countries can draw on the U.S. experience when considering how to regulate these products. Lessons learned from the U.S. also show the importance of assessing the unintended consequences of certain policies or lack of regulations. In addition, the tobacco industry might exploit policy wins in the U.S. to advocate for the introduction of its products in other countries [60]; given that this is a global industry, information from a specific country might be useful to inform efforts in other countries. Moreover, the use of a framework to classify the literature is a strength of the study, because it informs critical gaps in the literature and highlights needs for the types of future studies to eliminate tobacco-related disparities and support a health equity research agenda.

### Implications for research, policy, and practice

Despite marketing being widely recognized as a driving force of disparities in tobacco use, studies in this review were categorized as second generation according to the HEART paradigm; that is, they sought to determine causal relationships that underlie disparities [15]. However, studies lacked recognition and discussion of factors other than advertising that influence one’s health. For example, studies examining e-cigarette or HTP advertising bans should apply an intersectional approach that analyzes these sociodemographic factors within the historical, social, political, cultural, and regulatory context [61] to elucidate the underlying factors that result in health inequities and ways of eliminating them. Overall, studies should be mindful of sample size to have enough statistical power to detect and monitor disparities across groups and subgroups. Further, future research should go beyond evaluating disparities, and address the third and fourth generations of the HEART paradigm which aim to evaluate and develop multilevel interventions and take action to achieve health equity. While cross-sectional studies such as those in our sample support the investigation of exposures and outcomes, they do not allow for determining causality and only represent a point in time; mixed-methods research provide a robust methodology to support the assessment of multilevel interventions [15]. To foster this critical research to be undertaken, research funding streams should encourage and prioritize health equity research, including community based participatory research, and increase involvement and leadership from researchers of diverse backgrounds. Moreover, it is fundamental that researchers work in partnerships with governments and local communities on eliminating health inequities.

The potential benefits of e-cigarettes as a cessation aid and heated tobacco products as a less toxic product compared to cigarettes are yet to be confirmed. Still, these are harmful products, especially for those who have not previously initiated any tobacco use, and their regulation has presented challenges to governments around the world. Therefore, it is essential to implement strong restrictions to tobacco advertising and promotion in order to avoid unintended consequences such as youth uptake [62, 63]. It is also important to consider the impact of any proposed policy on the basis of the multiple factors discussed here to avoid increasing or maintaining tobacco-related disparities even if tobacco use is reduced overall.

## Conclusions

This study showed that e-cigarette and HTP advertising and promotion varies based on several sociodemographic characteristics; given that the potential benefits of these products would be restricted to current smokers and youth were more likely to be exposed to advertising, tobacco-related disparities might further increase. Moreover, this study highlights the need for research explicitly incorporating and applying a health equity lens from its inception in order to obtain data that can support the elimination of tobacco-related disparities.

## Data Availability

All data analyzed during this study are included in this published article.

## Abbreviations

HEART: Health Equity Action Research Trajectory
HTP: Heated tobacco product
POS: Point-of-sale
SES: Socioeconomic status
